# Endoscopic posterior ventricular cordectomy with contact diode laser: how I do it

**DOI:** 10.1007/s00405-023-08234-z

**Published:** 2023-09-29

**Authors:** Stefano Meneghesso, Roberto Saetti, Marina Silvestrini

**Affiliations:** 1 Otorhinolaryngology-Head and Neck Department, University Hospital of Verona, Verona, Italy; 2 Department of Otolaryngology, Ospedale San Bortolo, Vicenza, Italy

**Keywords:** Bilateral vocal fold paralysis, Laryngospasm, Posterior cordectomy, Endoscopic laryngeal surgery, Contact diode LASER

## Abstract

**Background:**

The treatment of bilateral vocal fold paralysis is mainly surgical and several procedures can be used to guarantee adequate breathing. Furthermore, other causes of the narrowing of the natural airways could coexist and the treatment should consider all of them.

**Methods:**

A supraglottic extension of posterior cordectomy to the false homolateral chord is described, which provides a further widening of the airway while maintaining acceptable voice quality.

**Conclusion:**

Endoscopic posterior ventricular cordectomy performed by contact diode laser may be a viable and safe option, especially in those patients who present bilateral vocal fold paralysis associated with various degrees of laryngospasm.

**Supplementary Information:**

The online version contains supplementary material available at 10.1007/s00405-023-08234-z.

## Background

Vocal fold paralysis (VFP) is a neurological condition that is characterized by reduced or absent movement of one or both vocal folds. When bilateral VFP (BVFP) occurs, both vocal folds assume a paramedian position, thus causing narrowing of the airway at the glottic level and therefore inspiratory dyspnea. VFP is mostly caused by laryngeal or extra-laryngeal cancers, iatrogenic injury during neck, thyroid gland, or chest surgery, and various neurogenic conditions. Patients with BVFP usually are recognized by noisy inspiratory breathing, with normal or nearly normal voice [1].

Treatment for BVFP is mainly surgical, and several procedures can be used. The most common one is tracheostomy, which is very effective but results in an open wound that requires continual postoperative management, and thus patients experience decreased quality of life [2].

Cordotomy is another common treatment for BVFP. It is an irreversible surgical procedure that results in airway enlargement at the glottic level. This is achieved with the excision of laryngeal soft tissues, such as parts of the vocal fold, the vocal ligament, or the thyroarytenoid muscle. Some authors describe this technique including the excision of the overlapping segment of the false vocal fold too [3]. Dennis and Kashima introduced the endoscopic laser cordotomy technique in 1989 [4]. This procedure has different advantages, such as rapidity, simplicity in concept, immediate assessment of the airway, reliability, short hospitalization, and low risk of complications. Cordotomy is susceptible to granulation and scar formation. It was reported that bilateral or revision cordotomies were needed in about 30% of patients due to a decreased glottal opening from the formation of scar tissue or granulation. Another most commonly seen side effect associated with cordotomy is the deterioration of voice quality. Patients often complained of a rough and breathy voice because of damage to the vibratory part of the operated vocal fold [5]. For these reasons, multi-stage surgery may be preferable. The basic goal of the procedure is to avoid tracheostomy with acceptable vocal impairment.

Depending on the case, the operation can be modulated from the simple section of the vocal ligament (*cordotomy*) to the more or less extensive removal of the posterior portion of the vocal muscle (*cordectomy*) to the lateral extension favored by the partial removal of the ventricular fold (*ventricular cordectomy*) [6].

In our experience, it is preferable to preserve at least the anterior two-third of the glottic plane to maintain acceptable vocal quality and to avoid sacrificing the arytenoid so as not to cause swallowing dysfunction. In selected cases, bilateral treatment may be considered at a later stage.

Several other procedures were studied to treat BVFP such as arytenoidectomy and laterofixation of the arytenoid and/or the attached vocal fold using a combination of endoscopic and external means. Even newer approaches are being developed, such as reinnervation, laryngeal pacing, gene therapy, and stem cell therapy in order to preserve the voice. However, there is very little clinical data about these new treatment options [2].

The technique described is a posterior cordectomy with supraglottic extension to the false homolateral chord. We chose this procedure in order to manage a BVFP affecting an 84-year-old female patient, who presented herself at our emergency ward for sudden dyspnea. She had recently undergone a left pulmonary apical lobectomy due to an adenocarcinoma. Fibrolaringoscopy evaluation showed a left paramedian vocal fold paralysis and an idiopathic hypomobility of the contralateral vocal fold, thus causing an important narrowing of the respiratory space. Furthermore, a relevant supraglottic laryngospasm with false vocal fold dystonia contributed to exacerbating her clinical status.

## Relevant surgical anatomy

Endoscopic laryngeal surgery essentially depends on exposure and on the clearest visualization of the glottic surface. A contribution to its evaluation comes from the Laryngoscore, in which several anatomical cranio-facial-laryngeal characteristics are studied. In endoscopic posterior ventriculo cordectomy, a central role is played by two laryngeal structures: vocal folds and ventricular or “false” folds. The former consists of the vocal ligament, with the overlying lamina propria and epithelium, and the vocalis muscle, which is namely the thyroarytenoid muscle. The latter is the inferior edge of the quadrangular membrane, a sheet of connective tissue and its mucosa which is attached superiorly to the lateral aspect of epiglottis. The space between the superior surface of the true vocal folds and the ventricular fold is Morgagni’s ventricle. In surgical terms, it is relevant to consider the ventricular fold volume as well as the extension of the ventricle’s depth, as it well predicts the extent of its opening until the perichondrium of the thyroid cartilage is reached and the underlying vocal fold is entirely visible.

## Description of the technique

**Fig. 1:**
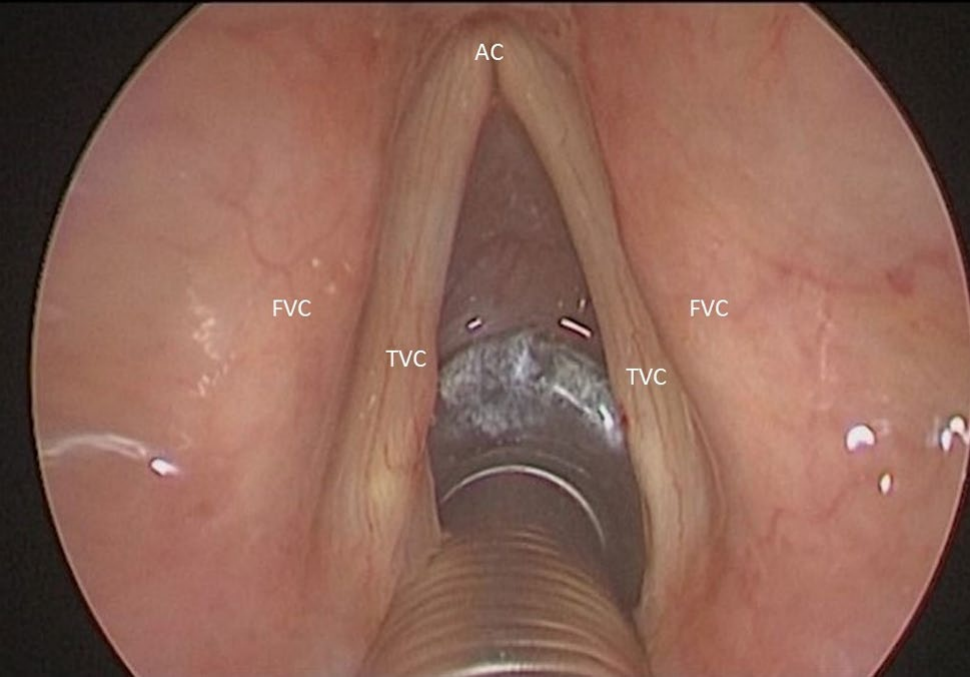
Endoscopic view of the glottic surface. *TVC* True Vocal Cord, *FVC* False Vocal Cord, *AC* Anterior Commissure

**Fig. 2:**
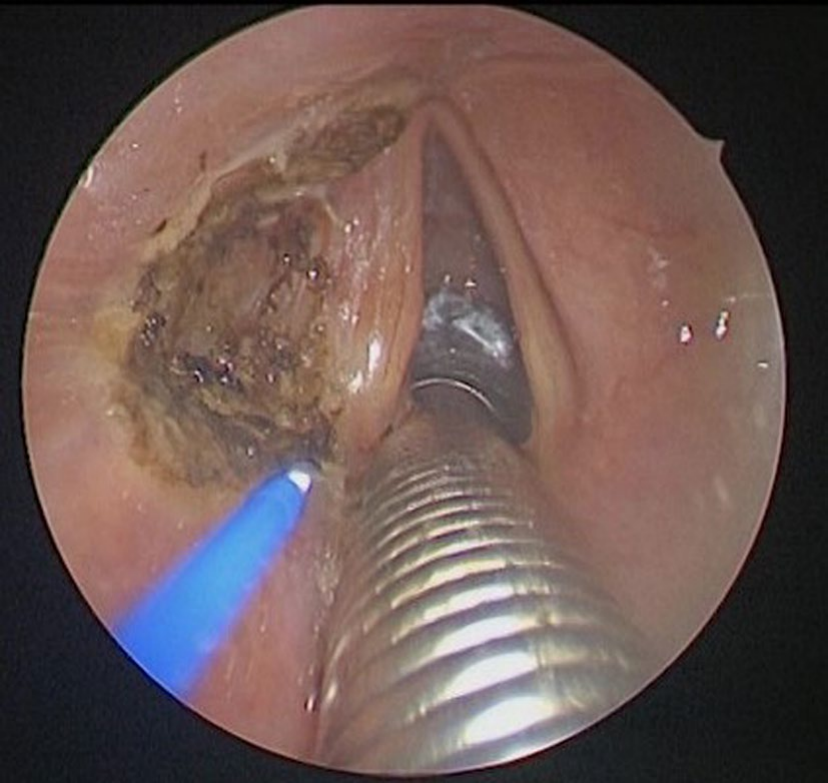
Incision of the left ventricular fold

**Fig. 3:**
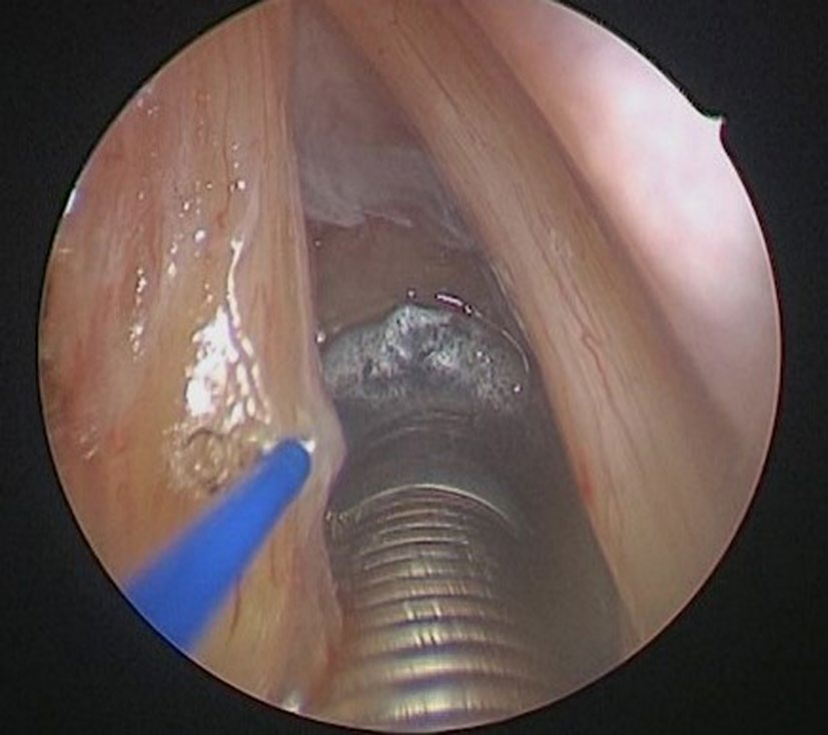
Left cordectomy, incision in the posterior third of the vocal fold

**Fig. 4:**
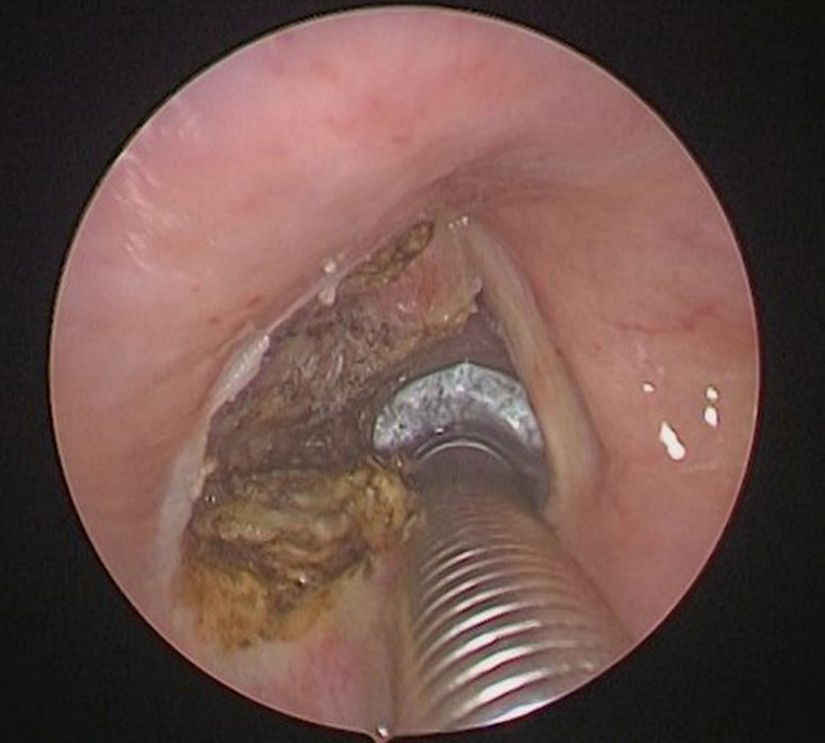
Endoscopic view of the glottic surface at the end of the left ventricular cordectomy

The procedure is performed under general anesthesia. Due to the narrowed airway which characterizes these patients, endoscope-guided intubation is usually undertaken with the positioning of a laser-protected tube. After proper relaxation, the larynx is exposed with a Lindholm laryngoscope, which is held in place by a laryngoscope chest holder once the glottis is clearly visible. A Bettocchi 5 mm operative hysteroscope allows inspection of the area with a 0° 4 mm videoendoscope (Fig. 1). This device enables the contact diode laser and the optic strictly connected on line and to suck fog or instill different solutions at the same time through two other working channels fitted for semi-rigid operative instruments. More in detail, contact diode laser has a 600 µm cone-shaped vectorial fiber tip which allows greater thermal diffusion to neighboring tissue applying the same intensity as CO_2_ laser; hence, the power should be usually set on average 10W, in order to attain the right balance between cutting precision and thermal coagulation. The first step of posterior ventriculo-cordectomy is the excision of the ventricular fold (Fig. 2). Resection begins between the medial and anterior thirds of the free margin of the ventricular fold with a transverse incision of the mucosa and then continues laterally and posteriorly until thyroid cartilage is reached. A sail-shaped portion of the fold is thus removed leading to an opening of Morgagni’s ventricle and unveiling the superior aspect of the vocal fold entirely. Cordectomy is then performed cutting a 3.5–4 mm wedge in the posterior third of the vocal fold (Fig. 3). The incision is made full-thickness starting from the free edge of the vocal fold and then extending it postero-laterally until it reaches the anterior part of the vocal process of the arytenoid. The mucosa of the vocal process is then vaporized by laser, taking care not to expose the underlying cartilage. At the end, a transverse opening at the level of the mid-posterior larynx is created, which is wider than a traditional cordectomy due to the previous overlying ventriculectomy (Fig. 4). The whole lased area is finally cleansed with cold saline solution and checked for additional hemostasis, if necessary.

## Indications and limitations

Tracheostomy still remains the most effective management for bilateral fold paralysis, but posterior cordectomy is another effective option. The decision should be evaluated considering the patient's individual needs and the surgeon’s preference. Patients must understand that to improve their airways the quality of their voice may be adversely affected. A contraindication to posterior cordotomy is the presence of aspiration or a rapidly progressive neurologic disorder. An important limitation is the uncertainty of a good exposition of the glottic surface that could affect the possibility of performing this procedure and the risk, in case of glottic stenosis or persistence of dyspnea, of a re-intervention.

## How to avoid complications

Possible intraoperative complications are hemorrhage and edema due to the incision, which could also limit the view of the surgical field and compromise the respiratory outcome. The possibility of using LASER, in particular contact diode one, allows good hemostasis and surgical precision with limited thermal damage resulting in decreased intraoperative and postoperative edema. During the procedure, it is important to avoid the exposition of arytenoid cartilage to prevent the formation of granulation tissue and preserve arytenoid and inter-arytenoid mucosa to avoid aspiration or posterior glottic stenosis. In order to prevent the formation of a postoperative cordal synechia, it is also important to perform the excision only on one side and avoid mucosal damage of the contralateral side. For good healing of the surgical site, patients should receive perioperative proton pump inhibitor medication to decrease exposure to reflux contents.

## Summary

1. Bilateral vocal fold paralysis (BVFP) is characterized by reduced or absent movement of both vocal folds causing narrowing of the airway.
2. The treatment of bilateral vocal fold paralysis is mainly surgical.
3. Cordotomy is an irreversible surgical procedure that results in airway enlargement at the glottic level.
4. Cordotomy has different advantages such as rapidity, simplicity in concept, immediate assessment of the airway, reliability, short hospitalization, and low risk of complications.
5. A common side effect associated with cordotomy is the deterioration of the quality of voice.
6. The basic goal of the procedure is to avoid tracheostomy with acceptable vocal impairment.
7. It is possible to extend the cordotomy to the ventricular fold (ventricular cordectomy) in case of dysfunction of the supraglottis.
8. It is preferable to preserve at least the anterior two-third of the glottic plane to maintain acceptable vocal quality and to avoid sacrifice of the arytenoid so as not to cause swallowing dysfunction.
9. Endoscopic laryngeal surgery essentially depends on exposure and on the clearest visualization of the glottic surface.
10. The use of contact diode LASER allows good hemostasis and surgical precision with limited thermal damage resulting in decreased intraoperative and postoperative edema.

### Supplementary Information

Below is the link to the electronic supplementary material.

Supplementary file1 (MP4 303592 KB)
